# Artesunate, as an Hsp90 inhibitor, inhibits the proliferation of Burkitt’s lymphoma cells by inhibiting AKT and ERK

**DOI:** 10.3389/fphar.2023.1218467

**Published:** 2023-08-31

**Authors:** Li Yuan-Ce, Zhang Qi, Zhang Hong-Yang, Wang Yan-Wen, Sun Yu-Mei, Yang Bi-Juan, Yin Jun-Lin

**Affiliations:** Key Laboratory of Chemistry in Ethnic Medicinal Resources, State Ethnic Affairs Commission and Ministry of Education, School of Ethnic Medicine, Yunnan Minzu University, Kunming, China

**Keywords:** artesunate, Hsp90, client protein, lymphoma, PLGA-PEG

## Abstract

**Introduction:** Artesunate, a derivative of artemisinin, has anti-malarial effects, and in recent years has also been reported to have anti-tumor activity. However, its anti-tumor mechanisms are not well understood.

**Methods:** In this study, we focused on the targeting of Hsp90 by artesunate to inhibit tumor cell proliferation, which we examined using immunoprecipitation, a proliferation assay, flow cytometry, western blotting, a tumor xenograft animal model, and immunohistochemistry. Furthermore, to examine the tumor-suppressive effects of artesunatein nude mice, we used artesunate-loaded PLGA-PEG nanoparticles.

**Results:** The binding of artesunate to Hsp90 was found to reduce the expression of its client proteins AKT, ERK, p-AKT, p-ERK, and EGFR, thereby blocking the cell cycle at the G0/G1 → S stage in lymphoma cells and inducing apoptosis. In addition, the results of tumor xenograft experiments revealed that artesunate reduced the expression of AKT and ERK proteins in tumor tissues, inhibited tumor proliferation, and reduced tumor size and weight. Furthermore, nanoparticle encapsulation was demonstrated to enhance the anti-cancer activity of artesunate.

**Discussion:** We thus established that artesunate inhibits the proliferation of lymphoma cells by targeting the Hsp90 protein, and we accordingly believe that this compound has potential for development as a novelanti-tumor drug.

## 1 Introduction

Burkitt’s lymphoma is an aggressive type of tumor that originates in the lymphatic system and is difficult to treat, thereby posing a serious threat to patients’ lives. Its complex etiology leads to a rapid onset of lesions and is associated with low cure rates (Schmitz et al., 2012; López et al., 2022). The current clinical treatment for this cancer is rituximab combined with CHOP (adriamycin, vincristine, prednisone) (Wang et al., 2019), which is associated with a 4-year post-treatment survival of between 60% and 70%. However, approximately 30% of patients develop drug resistance and some patients experience tumor metastasis (Crombie and LaCasce, 2021). Consequently, there is a pressing need to develop novel anti-cancer drugs.

Artesunate, a hydrophilic derivative of artemisinin, is a clinical drug that is generally used for the treatment of cerebral malaria (Tu, 2011). In recent years, however, artemisinin-related derivatives have also attracted increasing attention in the fields of anti-inflammatory (Lei et al., 2021), anti-cancer (Zhao et al., 2020), immunosuppressive (Krishna et al., 2008) therapy. According to the findings of recent relevant studies, artesunate can be administered to treat a range of tumor types, including leukemia, colon cancer (Jiang et al., 2018), melanoma (Berköz et al., 2021), breast cancer (Pirali et al., 2020), ovarian cancer (McDowell et al., 2021), prostate cancer (Vakhrusheva et al., 2022), brain cancer (Kong et al., 2019), and renal carcinoma. In this context, drug target identification has become an increasingly active area of research, and in-depth studies of drug-to-target effects can contribute to enhancing our understanding the mechanisms underlying drug activity. Interestingly, artesunate can induce apoptosis in non-Hodgkin’s lymphoma (Vatsveen et al., 2018) and downregulates the expression of cell cycle protein-dependent kinase 4 (CDK4) expression, thereby blocking the cancer cell cycle (Tran et al., 2014).

Heat shock protein 90 (Hsp90), a member of the heat shock protein family, is a homodimer, the expression of which is induced by stress responses in tissues (Schopf et al., 2017). This members of this protein family plays essential roles in maintaining the stability of downstream proteins. The so-called “client proteins” of Hsp90 include protein kinase B (AKT), p53, epidermal growth factor receptor (EGFR), Raf, and mitogen-activated protein kinase kinase (MEK) (Isaacs, 2016). In cancer cells, the presence of these client proteins is inextricably linked with proliferation and apoptosis. Mutations or overexpression of client proteins may lead to tumorigenesis (Zeng et al., 2021). Previous studies have found that whereas the expression of Hsp90 in normal cells is generally low, in human tumor tissues it is activated in a disordered manner. In tumor cells, activated Hsp90 influences the physiological functions of client proteins such as AKT, ERK, and EGFR, thereby enhancing their stability and levels of expression. Among these proteins, AKT and ERK are associated with cell cycle arrest and apoptosis, whereas EGFR influences tumor cell proliferation by interfering with vascular cell metastasis. Inhibitors of Hsp90 kill tumor cells by synergistically interfering with multiple signaling pathways (Sabbah et al., 2020), and the findings of numerous studies have indicated that these HSP90 inhibitors are therapeutically effective against a range of tumors types, including leukemias, colon cancer, melanomas, breast cancer, ovarian cancer, and prostate cancer. Notably, several inhibitors of Hsp90 have entered clinical trials, among which are tanespimycin and 17-AAG. Accordingly safe and effective Hsp90 inhibitors that have inhibitory effects against a range of cancer types in preclinical or clinical trials are likely to have considerable market potential.

Poly (lactic acid-co-glycolic acid)–poly (ethylene glycol) (PLGA-PEG) is a polymeric material routinely used for drug delivery (Doan et al., 2022) that degrades to lactic and glycolic acids in the tricarboxylic acid (TCA) cycle, and causes minimal side effects in the human body (Duan et al., 2021). Compared with materials previously used for drug delivery, such as lactose, PLGA-PEG has superior solubility (Wang et al., 2019), and is more readily fabricated as macromolecular microspheres, and has thus been more widely used in drug delivery. PLGA-PEG can be used to encapsulate drugs to form nanoparticles, which can accumulate in tumor tissue in response to the action of EPR, and therein gradually release the encapsulated drug upon degradation of the PLGA-PEG shell.

However, it has yet to be determined whether targeting Hsp90 with artesunate would influence the stability of AKT and ERK and thereby inhibit the proliferation of Burkitt’s lymphoma cells. In this study, we demonstrated that the binding of artesunate to Hsp90 reduces the expression of its client proteins AKT, ERK, p-AKT, p-ERK, and EGFR, thereby blocking the G0/G1 phase → S phase of lymphoma cells and inducing apoptosis. Moreover, we established that nanoparticle encapsulation can enhance the anti-cancer efficacy of artesunate. Our findings in this study provide new insights into the anti-tumor mechanisms of artesunate.

## 2 Materials and methods

### 2.1 Experimental reagents

Streptavidin, a Protein Silver Stain Kit, and a Cell Cycle and Apoptosis Analysis Kit (PI staining) were purchased from Yeasen Biotech Co., Ltd. (Shanghai, China). Lymphoma cells used in experiments (SU-DHL-4, Daudi, CA-46, and Jeko-1) were purchased from the Kunming Cell Bank of the National Science Academy of China. (Kunming, China). A BrdU Cell Proliferation ELISA Kit was purchased from abcam-Innovating Technology. (Cambridge, UK). Artesunate (98%) was purchased from Aladdin (Shanghai, China) Primary antibodies (AKT, p-AKT, ERK, p-ERK, EGFR, *β*-actin, PARP, caspase 3, cleaved-caspase 3, caspase 9, cleaved-caspase 9, CDK1, CDK4, and CyclinB1) and secondary antibodies (rabbit and mouse) were purchased from Proteintech (Wuhan, China).

### 2.2 Synthesis of the AP-1 probe

A mixture of artesunate (1 mmol), mono-propargylamine (1.2 mmol) and TEA (2 mmol) in CH_3_CN (3 mL) was stirred at 0°C for 10 min. HATU (1.2 mmol) was then added in one port, and the reaction was carried out under nitrogen protection. After 3 h, the CH_3_CN was removed *in vacuo* and the residue was purified by running on a silica gel column chromatography (X. Zhang et al., 2022). The yield of AP-1 was 84%. Nuclear magnetic resonance (NMR): ^1^H NMR (400 MHz, chloroform-*d*) *δ* 6.01 (s, 1 H), 5.78 (d, *J* = 9.8 Hz, 1 H), 5.44 (s, 1 H), 4.23–3.75 (m, 2 H), 2.95–2.68 (m, 2 H), 2.66–2.44 (m, 3 H), 2.38 (td, *J* = 14.1, 3.9 Hz, 1 H), 2.23 (t, *J* = 2.6 Hz, 1 H), 2.03 (ddd, *J* = 14.6, 4.9, 3.0 Hz, 1 H), 1.89 (ddd, *J* = 13.4, 6.4, 3.3 Hz, 1 H), 1.75 (ddq, *J* = 23.3, 16.5, 3.5 Hz, 4 H), 1.63 (dt, *J* = 13.8, 4.5 Hz, 1 H), 1.59–1.44 (m, 1 H), 1.32 (dddd, *J* = 25.7, 17.4, 12.3, 5.0 Hz, 3 H), 1.13–0.99 (m, 2 H), 0.97 (d, *J* = 5.8 Hz, 3H), 0.85 (d, *J* = 7.1 Hz, 3 H). ^13^C NMR (100 MHz, CDCl_3_) *δ* 171.7, 170.9, 104.5, 92.2, 91.5, 80.1, 71.6, 51.5, 45.2, 37.3, 36.2, 34.1, 31.8, 30.6, 29.6, 29.3, 26.0, 24.6, 22.0, 20.2, 12.1.

### 2.3 Cell thawing

Cells were removed from liquid nitrogen storage and thawed in a water bath at 37°C. The cells were then resuspended in 2 mL of RPMI1640 medium and the suspension was centrifuged at 800 rpm and 4°C for 5 min. Having removed the supernatant, the cells were resuspended in 10 mL of RPMI1640 (high glucose) and placed in a 37°C, 5% CO_2_, humidified incubator.

### 2.4 Cell passaging

The cell cultures were centrifuged at 800 rpm and 4°C for 5 min, and having removed the supernatant, the pelleted cells were resuspended in 2 mL RPMI1640 medium. A 200-µL aliquot of this suspension was used to inoculate 10 mL of culture medium and incubated in a 5% CO_2_ humidified incubator at 37°C.

### 2.5 Docking analysis

Crystal structure data for HSP90 and ligand (ID: 6cyg) were downloaded from the Protein Data Bank. The intrinsic ligand and water molecules in 6cyg were removed using PYMOL2.2 software. Artesunate minimum energy optimization was performed using ChemDraw3D software, and polar hydrogens were added using Tools-1.5.6 software. Two relative positions were defined using MarvinSketch software (Chaturvedi et al., 2010), namely, the radical formed by peroxide bridge cleavage and the electronegative amino acid located in the pocket. The localization parameters were imported into MOE software, treating the intrinsic coordination cavities as binding pockets, and on the basis of this localization file and algorithm, tMOE software was used to performing docking, which was analyzed using Schrödinger.

### 2.6 Proliferation assay using the sulforhodamine B method

Cells, artesunate, and DMSO were added to cell culture plates at concentrations of 20, 10, 5, and 2.5 μg/mL (artesunate) and 0.1% (DMSO) and incubated for 24 h. After incubation, the cells were fixed with 25 µL of 80% trichloroacetic acid for 2 h. Each well was stained with 100 µL of 4% sulforhodamine B solution for 30 min. After treatment with 100 µL of TRI lysate, the plates were shaken for 15 min and measurements were obtained using a 550-nm microplate reader.

### 2.7 Cell proliferation assay using the BrdU method

Cell cultures were centrifuged at 200 × *g* for 5 min, and following removal of the supernatant, 200 µL of fixative was added to each well of plates for 30 min followed by three washes with phosphate-buffered saline (PBS). Following the addition of 100 µL of probe antibody to each well, plates were incubated for 1 h at room temperature and again washed three times with PBS. Thereafter, 100 µL of horseradish peroxidase-conjugated secondary antibody was added to each well, followed by incubation for 30 min. Having washed three times with PBS, 100 µL of TMB was added to each well to stop the reaction and measurements were performed using a 550-nm UV detector.

### 2.8 Immunoprecipitation assays

Following lysis, proteins were extracted from AP-1 cultured cell. To the extracted protein suspension, we added 1 µL of biotin azide (2,500 µM), 1 µL of TCEP (500 mM), 1 µL of TBTA (50,000 µM), and 500 mM CuSO_4_, followed by dilution with buffer solution and mixing. The diluted solution was incubated for 3 h at 4°C, and after completion of the reaction, the mixture was centrifuged at 12,000 × *g* for 10 min at 4°C, and thereafter washed three times with acetone. Having allowed to dry at room temperature, 500 µL of 2.5% SDS in PBS was added to the protein preparation followed by boiling at 100°C for 5 min. The suspension was then centrifuged and the supernatant was transferred to a fresh centrifuge tube, to which 60 µL of resin was added followed by mixing for 3 h. The mixed suspension thus obtained was then centrifuged at 1,000 × *g* for 10 min. Having removed the supernatant, the pellet was washed with PBS, followed by the addition of loading buffer, diluted with PBS, and then boiled at 100°C for 10 min. The expression of proteins in the lymphoma cells was analyzed using western blotting.

### 2.9 Apoptosis assays

Cells were collected in 1.5-mL Eppendorf tubes and centrifuged at 300 × *g* for 5 min at 4°C. The pelleted cells were washed twice with PBS, followed by the addition of 100 µL of 1× binding buffer. Following thorough mixing, 5 µL of Annexin V-FITC and 10 µL of PI staining mixture were added to the cell suspension, which was then placed in the dark for 15 min. Having added 400 µL of 1× binding buffer. PI staining was measured using Annexin V-FITC flow cytometry.

### 2.10 Flow cytometric assay of cell cycle phase

Cells were collected by centrifuging at 1,000 × *g* for 5 min. Having discarded the supernatant, the cells were resuspended in pre-cooled 75% ethanol and fixed overnight at 4°C. Thereafter, the cells were centrifuged at 1,000 × *g* for 5 min and after discarding the supernatant, the sediment was washed once with PBS. Having prepared a mixture of staining buffer, containing 10 µL of propidium iodide and 10 µL of ribonuclease A, 0.5 mL of the PI staining buffer was mixed with the cells followed by incubation for 30 min at 37°C.

### 2.11 Western blotting

Cells were collected in 15-mL centrifuge tubes, centrifuged at 1,000 × *g* and 4°C for 5 min, and after discarding the supernatant, the resulting cell pellets were washed twice with PBS. Having added cell lysis buffer, the suspensions were heated at 98°C for 10 min, and aliquots of the resulting lysate supernatant were subjected to gel electrophoresis. The membranes were blocked with 5% skimmed milk for 2 h, and thereafter incubated overnight with primary antibodies at 4°C. The following day, having washed five times with TBST (each for 5 min), the membranes were incubated for 2 h with secondary antibody (1:7,000), followed by five 5-min washes with TBST. Gray-scale values were obtained using Image J.

### 2.12 Preparation of PLGA-PEG nanoparticles

An emulsion was initially prepared by adding 100 mg PLGA-PEG and 50 mg artesunate powder to 1 mL of CH_2_Cl_2_, which was then added dropwise to 30 mL of 2% PVA solution with continuous stirring. The resulting emulsion was treated with ultrasound at 300 W for 3 min and then stirred overnight with a magnetic stirrer at 150 rpm to obtain a suspension. Following centrifugation at 12,000 rpm for 10 min, the supernatant was discarded and the precipitate was freeze-dried.

### 2.13 A nude mouse subcutaneous xenograft model

To prepare a subcutaneous xenograft model, we used 4- to 6-week-old Male nude mice (BALB/c-nu) (16–20 g), obtained from Hunan SJA Laboratory Animal Co., Ltd. (Hunan, China). All male mice were maintained at room temperature (22°C–25°C) under a 12-h light/12-h dark cycle and given free access to food and water. During experiments, all mice remained in good health. All procedures involving animal experiments followed the Guide for the Management of Laboratory Animal Affairs and the Ethical Guidelines for Animal Experimentation System of Yunnan Minzu University (2021-050). Allogeneic subcutaneous transplants were administered via intratumoral injection 4 days after transplantation. Mice were randomly divided into one of the following five groups, each containing six mice: control (saline), PLGA-PEG 400 mg/kg, artesunate-PLGA-PEG 400 mg/kg, artesunate-PLGA-PEG 200 mg/kg, and artesunate (artesunate 40 mg/kg). The mice were administered continuously with e respective preparations for 21 days, and during this period, changes in body weight were recorded. After 21 days, tumor tissues was removed from the mice subcutaneously and difference between the groups with respect to tumor weight and volume were recorded.

### 2.14 Immunohistochemical analyses

Dehydrated tumor tissues were embedded in paraffin wax. Slices were prepared by heating in a water bath to recover the antigen. The slices were placed in a pressure cooker, heated for 10 min together with EDTA, and after gradually cooling to room temperature, were washed three times with PBS. Having blocked with 10% goat serum (used as a blocking antibody) for 1 h and washed times with PBS, the tissue slices were incubated overnight with the primary antibody (AKT 1:100 or ERK 1:50) and the following day were washed three times with PBS. Thereafter, the slices were incubated with a secondary antibody at room temperature followed by three 20-min washes with PBS. Having initially placed the slices in DAB chromogenic substrate solution for 5–10 min, they were stained with hematoxylin for 2 min, rinsed twice with distilled water, and dehydrated for 2 min.

### 2.15 Statistical analysis

The quantitative data obtained in this study are presented as the means ± SE. Analyses were performed using GraphPad Prism 8 software. Student’s *t*-test was used to determine the statistical significance of differences between the different experimental groups. *p < 0.05, **p < 0.01, ***p < 0.001.

## 3 Results

### 3.1 Validation of the AP-1 targeting of heat shock protein 90

**FIGURE 1:**
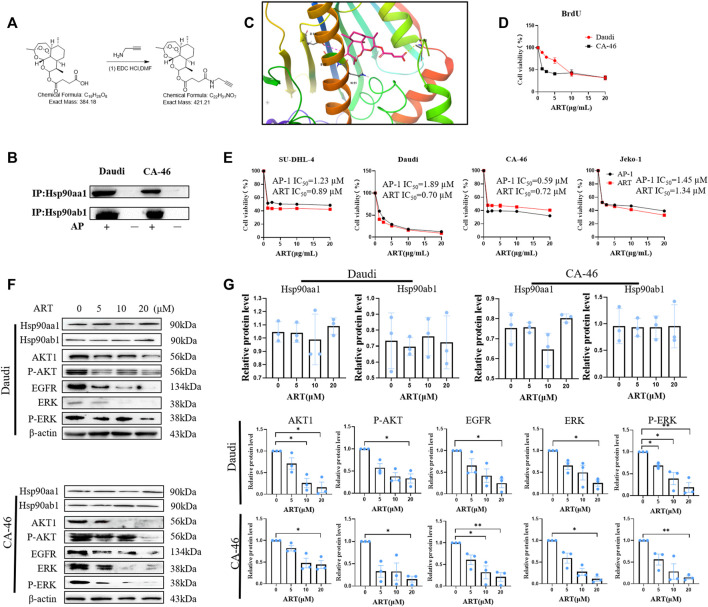
Artesunate targets HSP90 to suppress the proliferation of lymphoma cells. **(A)** The steps in the synthesis of the probe AP-1. **(B)** Immunoprecipitation assay of AP-1 and Hso90. 5 μM AP-1 was incubated with lymphoma cells for 24 h. **(C)** Analysis of covalent docking between artesunate and the Hsp90 protein; **(D)** BrdU assay performed to determine the inhibitory effects of artesunate on lymphoma cell proliferation. The effects of 20, 10, 5, and 2.5 µM artesunate (24 h) on CA-46 and Daud cells. **(E)** Inhibition of the proliferation of CA-46, Daudi, SU-DHL-4, and Jeko-1 cell lines by artesunate/AP-1 was detected using the SRB method. Black represents the AP-1 group and red represents the artesunate group. **(F)** The expression of the Hsp90 client proteins AKT, p-AKT, ERK, p-ERK, and EGFR measured using western blotting, after treatment with artesunate co-incubated with CA-46/Daudi cells for 24 h using artesunate dosages of 20, 10, 5, and 2.5 µM. **(G)** Analysis of protein gray-scale and normalization (*n* = 3. Data are presented as the means ± SEM. An independent Student’s *t*-test was used to determine the significance of differences. *p < 0.05, **p < 0.01, ***p < 0.001).

Daudi or CA-46 cells was co-incubated with AP-1. AP-1 penetrates the cell membrane, and on entering the cell cytoplasm and binds to Hsp90, thereby inhibiting cell proliferation. The binding of AP-1 to Hsp90 was assessed based immunoblotting. In the experimental group, we detected Hsp90aa1 and Hsp90ab1, whereas in the negative control group neither protein was detected, indicating that AP-1 binds to Hsp90, which has been identified as one of the targets of artesunate (Figure 1B).

### 3.2 Covalent binding of artesunate to Hsp90

We verified the binding between artesunate and Hsp90 using the AP-1 probe. On the basis of this validation, we conducted a preliminary examination of the binding pattern between artesunate and Hsp90 *in silico*. We hypothesized that covalent binding between artesunate and Hsp90 is induced by free radicals. With reference to the X-ray protein structure of Hsp90 in the Protein Data Bank, which was docked using the MOE method with a docking binding energy of −8.19 kcal/Mol, we used Schrödinger to analyze the covalent binding of artesunate to the ASP93 residue of Hsp90 and the binding mode of the salt bridge formed between Fe^2+^ and Ser52 (Figure 1C).

### 3.3 Inhibition of cell proliferation by artesunate and AP-1

The molecular probe AP-1 was obtained by covalently attaching an alkyne group to artesunate (Figure 1A). To reduce alkyne group interference, this group was located at a site other than the key bioactive site of artesunate. Subsequently, we compared the inhibition of malignant lymphoma cell proliferation by artesunate and AP-1 through experimental validation. In the BrdU assay, the survival of Daudi and CA-4 6 cells at an artesunate concentration of 20 µM was 31.91% ± 5.122% and 31.99% ± 3.88%, respectively (Figure 1D). Among the four assessed lymphoma cell lines SU-DHL-4, Daudi, CA-46, and JEKO-1, we obtained artesunate IC_50_ values of 0.89 ± 0.12, 0.70 ± 0.29, 0.72 ± 0.05, and 1.34 ± 0.31 µM, respectively, whereas the corresponding IC_50_ values for AP-1 were 1.23 ± 0.24, 1.89 ± 0.28, 0.59 ± 0.17, and 1.45 ± 0.28 µM (Figure 1E). These values thus indicate that the covalent linkage of alkynes to ART had no appreciable adverse effects on the inhibitory activity artesunate against cell proliferation.

### 3.4 Artesunate reduces the expression of Hsp90 client proteins

The effects of artesunate-induce inhibition on the expression of Hsp90aa1, Hsp90ab1, and associated client proteins (AKT, p-AKT, ERK, p-ERK, and EGFR) were investigated in the malignant lymphoma cell lines Daudi and CA-46 using western blotting. Artesunate (20 µM) was co-incubated with tumor cells and the relative levels of client proteins were measured in the negative control and experimental groups. Although we detected no significant changes in the level of Hsp90 following the treatment with artesunate, the relative levels of AKT, p-AKT, EGFR, ERK, and p-ERK proteins in the Daudi experimental group were found to be 16% ± 12%, 33.4% ± 10.4%, 24.3% ± 10.3%, 24.8% ± 7.1%, and 18.7% ± 11.3%, respectively, compared with those in the negative control group. Similarly, in the CA-46 experimental group, the relative contents of the corresponding client proteins were 44.2% ± 9.3%, 15.5% ± 6.5%, 21.2% ± 9.8%, 11.0% ± 5%, and 14.3% ± 3.6%, respectively. These findings accordingly reveal that the expression of AKT, p-AKT, EGFR, ERK, and p-ERK in the artesunate experimental group was significantly lower than that in the control group. (Figures 1F, G).

### 3.5 Artesunate induces apoptosis in malignant lymphoma cells

**FIGURE 2:**
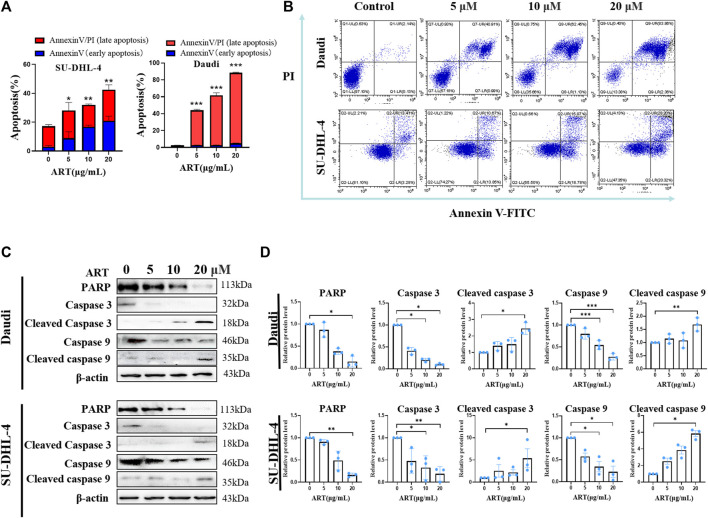
Artesunate induces apoptosis and changes in associated proteins. **(A)** Rates of lymphoma cell (SU-DHL-4/Daudi) apoptosis induced by artesunate administered at 20, 10, 5 and 2.5 µM for 24 h. The red portion represents the early apoptotic stage and the blue portion represents the late apoptotic stage. **(B)** The rate of apoptosis was determined using flow cytometry. The first quadrant represents late apoptosis, the second quadrant represents necrotic cells, the third quadrant represents surviving cells, and the fourth quadrant represents early apoptosis. **(C)** Expression of apoptosis-related proteins. **(D)** Gray-scale analysis of protein images. (*n* = 3. Data are presented as the means ± SEM. An independent Student’s *t*-test was used to determine the significance of differences. *p < 0.05, **p < 0.01, ***p < 0.001).

Artesunate-induced apoptosis was confirmed using flow cytometry, which revealed rates of early apoptosis of 0.76% ± 0.21%, 2.59% ± 0.44%, 2.70% ± 0.38% and 4.90% ± 0.72% in Daudi cells treated with artesunate at doses of 0, 5, 10, and 20 μM, respectively, whereas the corresponding values obtained for late apoptosis were 1.76% ± 0.17%, 41.41% ± 2.02%, 58.77% ± 5.41%, and 83.49% ± 1.16%. Comparatively, for the SU-DHL-4 cells, the early apoptosis rates were 3.05% ± 0.84%, 8.94% ± 4.44%, 16.73% ± 1.22% and 20.95% ± 3.24%, at artesunate doses of 0, 5, 10, and 20 μM, respectively, and the corresponding late apoptosis values were 14.17% ± 1.08, 19.13% ± 5.57%, 15.33% ± 0.54%, and 21.47% ± 3.39% (Figures 2A, B).

Compared with the negative control Daudi cells, the expression levels of PARP, caspase3, cleaved caspase3, caspase9, and cleaved caspase9 proteins in the 20 µM artesunate group were 15.3% ± 7.1%, 10.3% ± 1.3%, 245.3% ± 35.4%, 27.7% ± 4.3%, and 168.7% ± 25.5%. In the SU-DHL-4 cells, compared with the negative control cells, the expression levels of PARP, caspase3, caspase3, caspase9, and caspase9 proteins in the 20 µM artesunate group were 16.4% ± 2.5%, 18.9% ± 9.3%, 540.4% ± 211.5%, 22.6% ± 22.4%, and 583.2% ± 36.1%. These findings thus indicate that whereas the expression of PARP, caspase9, and caspase3 was significantly reduced in response to treatment with artesunate, the expression of cleaved caspase9 and cleaved caspase3 was significantly enhanced. On the basis of these findings, it can be deduced that in response to treatment with artesunate, the caspase9-caspase3 signaling pathway was activated to induce apoptosis in both the assessed lymphoma cell lines (Figures 2C, D).

### 3.6 Artesunate induces cell cycle arrest in malignant lymphoma cells

**FIGURE 3:**
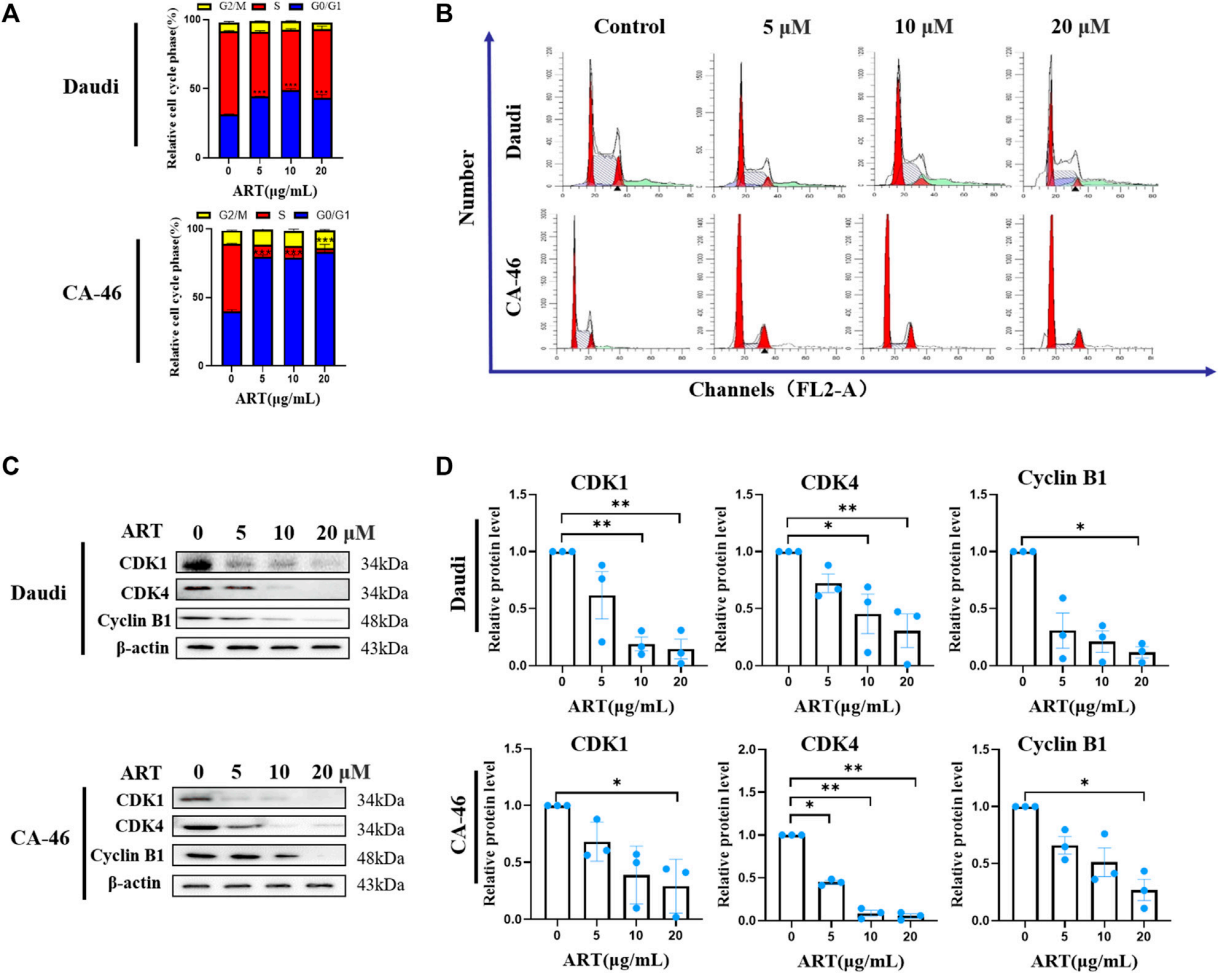
Lymphoma cell cycle arrest and expression levels of associated protein. **(A)** Proportional changes in each phase of the lymphoma cell cycle, in cells treated with 20, 10, 5 and 2.5 µM artesunate for 24 h. **(B)** Artesunate-induced lymphoma cell cycle arrest. The peak on the left side of the graph represents the G0/G1 phase, the shaded area in the center of the graph represents the S phase, and the second peak of the graph represents the G2/M phase. **(C)** Expression of cell cycle-related proteins. **(D)** Gray-scale analysis of protein images. (*n* = 3. Data are presented as the means ± SEM. An independent Student’s *t*-test was used to determine the significance of differences. *p < 0.05, **p < 0.01, ***p < 0.001).

Flow cytometric analysis verified that treatment with artesunate had the effect of blocking the lymphoma cell cycle. When administered at concentrations of 20, 10, 5, and 0 μM, we observed that the percentages of Daudi cells in the S-phase of the cell cycle were 49.67% ± 2.33%, 43.67% ± 0.67%, 46.67% ± 0.88%, and 60.33% ± 0.33%, respectively. Comparatively, the corresponding percentages of CA-46 cells in the S-phase were 2.66% ± 2.66%, 8.00% ± 1%, 9.00% ± 1.15%, and 49.00% ± 0.57%, respectively (Figures 3A, B). Compared with to the control group, the percentages of cells in the G0/G1 phase were significantly increased, whereas those in the S phase were significantly reduced following treatment with artesunate (Figures 3A, B). Western blot analysis, performed to assess the effects of artesunate on the expression of CDK1, CDK4, and Cyclin B1, revealed relative levels 14.7% ± 8.6%, 30.6% ± 14.8%, and 11.7% ± 4.8%, respectively, in Daudi cells, compared with the negative control, when administered at a concentration of 20 µM. Comparatively, the corresponding values in CA-46 cells treated with 20 µM artesunate were 29.2% ± 13.7%, 5.4% ± 2.5%, and 26.9% ± 16.3%, respectively. We thus established that in both the assessed lymphoma cell lines, the expression levels of CDK1, CDK4, and Cyclin B1 were reduced following treatment with artesunate (Figures 3C, D). Collectively, our experimental results thus provided evidence to indicate that artesunate can significantly inhibit the proliferation of lymphoma cells, in which the cell cycle distribution was altered and cells were arrested in the G0/G1 phase.

### 3.7 Tumor suppression in a nude mouse xenograft tumor model

**FIGURE 4:**
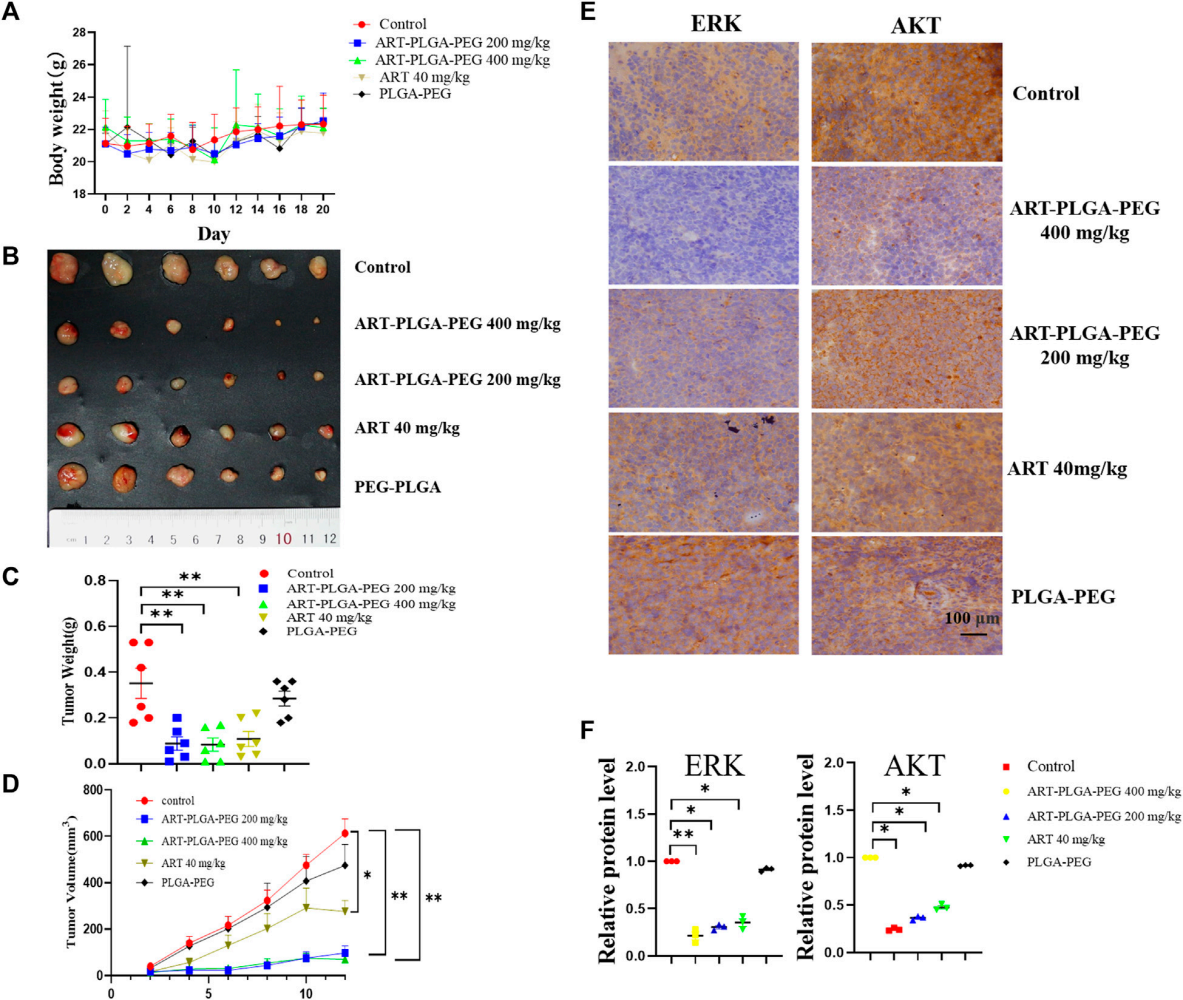
The subcutaneous xenograft lymphoma model. **(A)** Body weight. **(B)** A subcutaneous tumor derived from Daudi cells (1 × 10^7^ cells per mouse). Mice treated with artesunate/artesunate-PLGA-PEG (*n* = 6/group); **(C)** Tumor weights; **(D)** Tumor volumes. **(E)** Immunohistochemical analysis was performed to evaluate the expression of Hsp90 client protein, including AKT and ERK; **(F)** Gray-scale immunochemical analysis (Data are presented as the means ± SEM. An independent Student’s *t*-test was used to determine the significance of differences. *p < 0.05, **p < 0.01, ***p < 0.001).

Daudi cells were transplanted into the axillary tissues of nude mice, and 4 days later, the tumors were treated with drugs. Tumor size and morphology were observed, and 21 days later, tumor tissues were collected from mice and weighed. During the experiment, we detected no significant change in the body weight of the model mice (Figure 4A). On the basis of these findings and those reported previously in toxicological studies of artesunate (Wang et al., 2019; Chen et al., 2022), it can be assumed that artesunate can be safely administered to nude mice at this dose. In addition, we obtained tumor weights of 0.350 ± 0.06 g, 0.088 ± 0.03 g, 0.083 ± 0.03 g, 0.108 ± 0.03 g, and 0.285 ± 0.02 g for the control, artesunate-PLGA-PEG200 mg/kg, artesunate-PLGA-PEG400 mg/kg, artesunate, and PLGA-PEG200 mg/kg groups, respectively (Figures 4C, D). Compared with the control group, treatment with artesunate-PLGA-PEG 200 mg/kg, artesunate-PLGA-PEG 400 mg/kg, and artesunate reduced tumor volumes by 84.15%, 88.72%, and 55%, and tumor weight by 76.28%, and 69.14%, respectively. Accordingly, these findings revealed that both artesunate and artesunate-PLGA-PEG can inhibit the development of xenograft tumors *in vivo* (Figure 4B), and that the therapeutic effects are more pronounced when using PLGA-coated artesunate (Figures 4C, D).

We subsequently examine the expression of the heat shock protein client proteins ERK and AKT based on an immunohistochemical staining technique, with images being analyzed in gray-scale. With respect to ERK, we detected relative expression levels of 0.21 ± 0.07, 0.30 ± 0.03, 0.35 ± 0.07, and 0.91 ± 0.01 in the artesunate-PLGA-PEG400 mg/kg, artesunate-PLGA-PEG200 mg/kg, artesunate, and PLGA-PEG400 mg/kg groups respectively, which represent reductions of 79%, 70%, 65%, and 9%, respectively compared with the control group; The corresponding levels of AKT expression were 0.24 ± 0.01, 0.36 ± 0.01, 0.46 ± 0.02, and 0.91 ± 0.05, with respective reductions of 76%, 64%, 54%, and 9% compared with the control group. These findings thus indicate that artesunate can promote a significant inhibition of the expression of ERK and AKT in tumor tissues, and drugs packed within a PLGA-PEG coating showed significant tumor suppressive effects (Figures 4E, F).

## 4 Discussion

Within cells, the heat shock protein Hsp90 functions to maintain the structural stability of a diverse array of client protein, including AKT (Neckers, 2002), ERK (Lei et al., 2017), EGFR (Arteaga, 2003), CDK1, CDK4, and caspase9. To date, several agents that inhibit Hsp90 have been reported, among which are, tanespimycin (17-AAG) (Agnew et al., 2001), SNX-2112 (Okawa et al., 2009), NMS-E973 (Fogliatto et al., 2013), and STA-1474 (McCleese et al., 2009). However, only a few such agents have entered into clinical trials, and accordingly, identifying safe and effective Hsp90 inhibitors should be a focus of future drug development.

**FIGURE 5:**
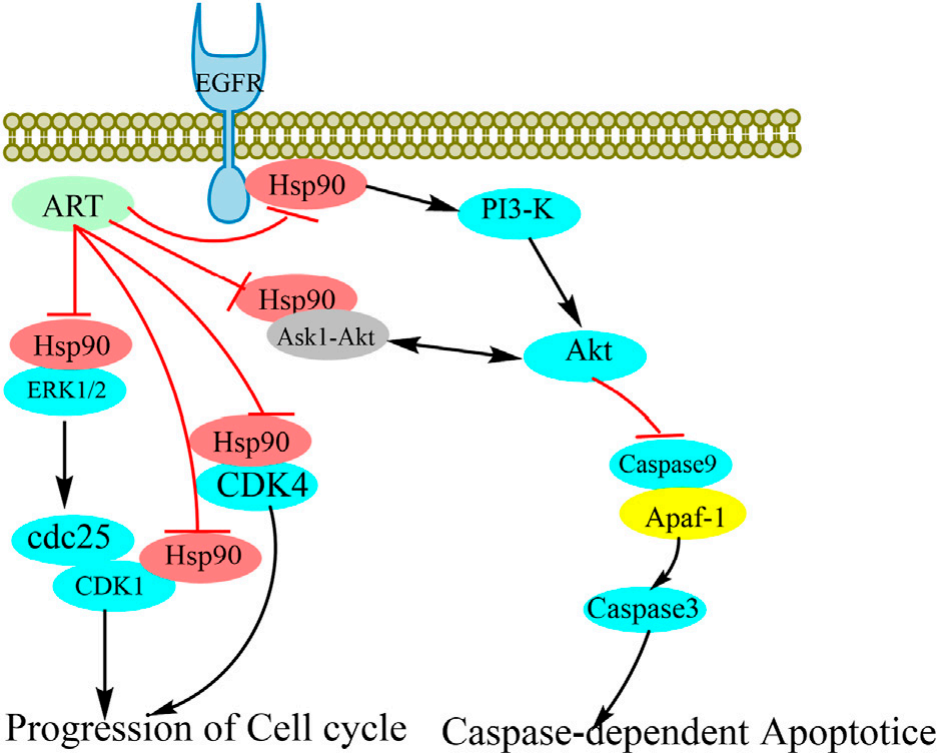
Schematic representation of the potential anti-tumor mechanisms of artesunate as an Hsp90 inhibitor. The stability of client proteins, including AKT, ERK, EGFR, CDK1, and CDK4, is influenced by Hsp90 binding. As a consequence of the inhibition of Hsp90 by artesunate, the interaction between Hsp90 and client proteins is disrupted, thereby influencing the stability and biological activity of the client proteins.

In recent years, artesunate has attracted considerable research attention on account of its notable anti-tumor activity. Numerous studies have shown that artesunate can act as an inhibitory agent against most tumor cell types, including breast cancer (Lai and Singh, 1995; Pirali et al., 2020), lung cancer (W. Zhang et al., 2022), bladder cancer (Zhao et al., 2020), ovarian cancer (McDowell et al., 2021), and thyroid cancer (Xu et al., 2022) cell lines. Given that the cancer-associated targets of artesunate have yet to be identified, in this study, we focused on the mechanisms whereby artesunate antagonizes lymphoma. To this end, we synthesized the AP-1 probe to identify the target proteins of artesunate, and accordingly found that AP-1 can bind to Hsp90aal and Hsp90ab1, thereby indicating that Hsp90 is among of the targets of artesunate. Notably, however, we established that artesunate causes neither an increase nor reduction in Hsp90 levels, on the bases of which, we examined correlation between artesunate and the client proteins of Hsp90s. In response to ART treatment, we detected reductions in the expression levels of a number of such proteins, including ERK, AKT, CDK1, CDK4, and caspase9. We further established that by inhibiting Hsp90, artesunate can reduce the stability of AKT, whereas it inhibits the activation of caspase9. Moreover, we found that a reduction in AKT content promoted apoptosis. In addition, it has previously been established that EGFR may activate the PI3K/AKT signaling pathway, activate CDK1 (Yang et al., 2022) and influence the cell cycle (Davis et al., 2014). In the present study, we found that the inhibition of Hsp90 by artesunate causes a reduction in the stability of EGFR, thereby reducing the contents of CDK1 and CDK4, which has the effect of blocking the cell cycle (Figure 5). These findings will accordingly provide a basis for the future development of artesunate as an anticancer drug.

However, despite its potentially promising effects, artesunate has the disadvantage of weak *in vivo* targeting and is rapidly metabolized (Li et al., 2005). To enhance the targeting properties artesunate and extend its metabolic stability in solid tumor tissues, we attempted to prepare drug nanoparticles using PLGA-PEG as a drug delivery material. The efficacy of these artesunate nanoparticles, which have an average size of approximately 100–200 nm (Supplementary Figures S5A–C), is dependent on the inherent EPR of PLGA-PEG, which facilitates the efficient accumulation in tumor tissues. Moreover, the encapsulated artesunate is less prone to hydrolysis than the prototype artesunate, thereby improving the stability of artesunate in water. Indeed, it has previously been demonstrated that compared with the artesunate prototype, artesunate nanoparticles have a more pronounced anti-lymphoma activity. Under the same experimental conditions, we recorded reductions in tumor weights of 88.72% and 55% in the artesunate-PLGA-PEG 400 mg/kg and artesunate treatment groups, respectively, thereby confirming that the encapsulation of artesunate can contribute to a greater than 30% increase in the efficacy of this agent.

However, despite our promising findings in this study, the study does have certain shortcomings. Notable among these is the fact that EGFR is closely associated with angiogenesis, and as yet, we have not undertaken an in-depth assessment of the consequences of the ART inhibition of EGFR activity via Hsp90 with respect to blood vessel growth. In the future, we will accordingly further refine our experimental approach. Moreover, given that artesunate has multiple active intermediates, it is not possible to accurately characterize the specific chemical binding mechanism between artesunate and HSP90. In the present study, we only briefly analyzed the plausible salt-bridge interactions between artesunate Fe^2+^ and the Ser52 residue of HSP90. Given that this is only one among a number of possible interactions between HSP90 and artesunate intermediates, we may at a later date further examine the interaction between the multiple active intermediates of artesunate and Hsp90.

## 5 Conclusion

In this study, we have succeeded in revealing the mechanisms underlying the activity of artesunate as an anti-tumor agent in the treatment of lymphomas. Specifically, by inhibiting HSP90, artesunate reduces the expression of its client proteins (AKT, ERK, and EGFR), thereby suppressing lymphoma cell proliferation. These findings provide new insights into the anti-tumor effects and mechanisms of artesunate.

## Data Availability

The original contributions presented in the study are included in the article/Supplementary Material, further inquiries can be directed to the corresponding authors.
