# Supplementary material for: Artesunate, as an Hsp90 inhibitor, inhibits the proliferation of Burkitt’s lymphoma cells by inhibiting AKT and ERK
Source: Front Pharmacol. 2023 Aug 31;14:1218467. doi: 10.3389/fphar.2023.1218467 (PMC10501146; doi:10.3389/fphar.2023.1218467)
Supplement: Supplementary file 1 [file DataSheet1.PDF]

Supporting Information for
ORIGINAL ARTICLE

Artesunate, as an Hsp90 inhibitor, inhibits the proliferation of Burkkit lymphoma cells by inhibiting AKT and ERK

LI Yuan-Ce , Zhang Qi , Zhang Hong-Yang , WANG Yan-Wen , Sun Yu-Mei , Yang Bi-Juan✉ , YIN Jun-Lin✉

Key Laboratory of Chemistry in Ethnic Medicinal Resources，State Ethnic Affairs Commission and Ministry of Education，School of Ethnic Medicine，Yunnan Minzu University，Kunming 650500，China；

Tel: +86 65946920

Email: yinjunlin1979@sina.com

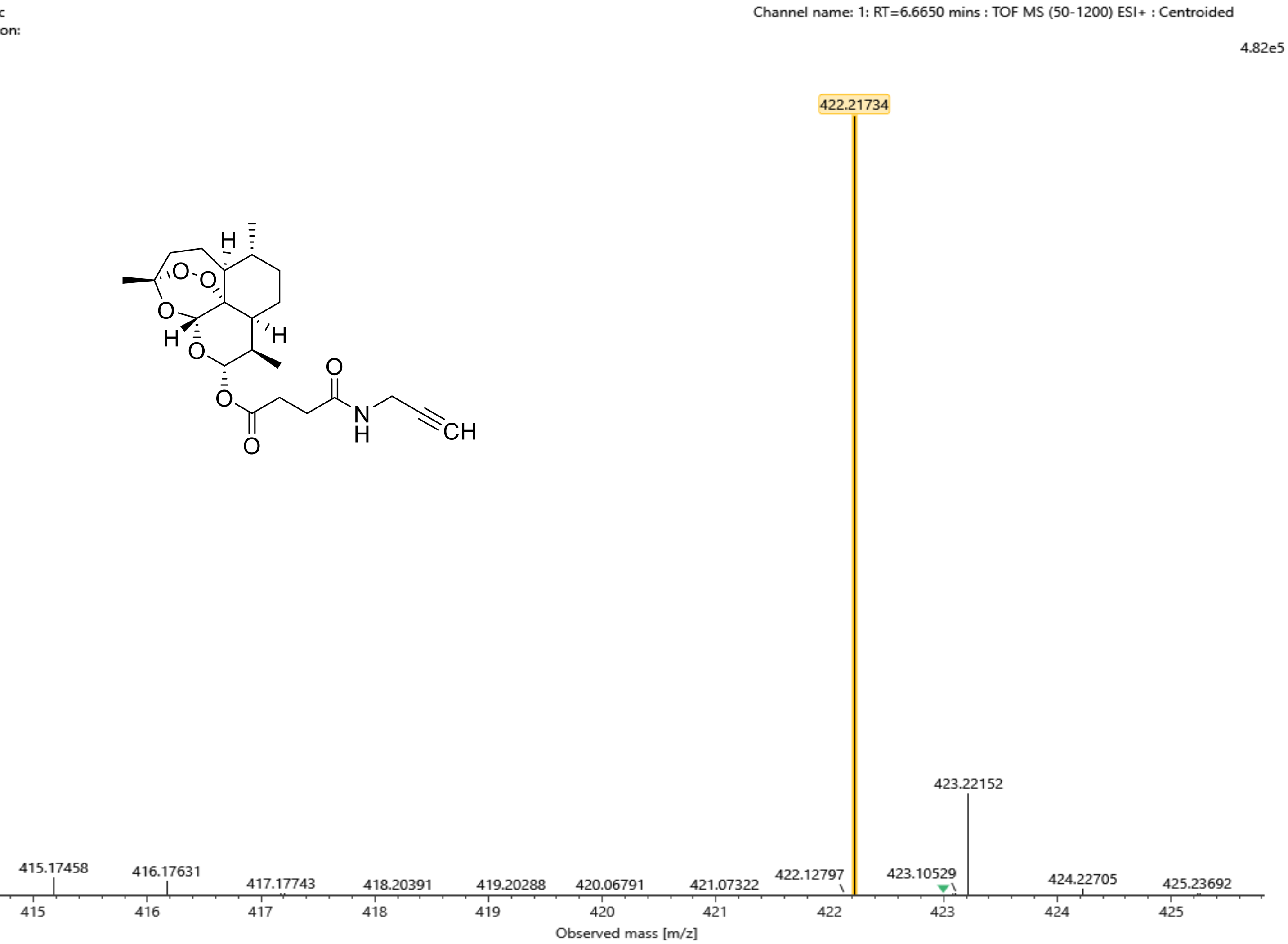

Fig S1 HRMS spectrum of PHY-PE

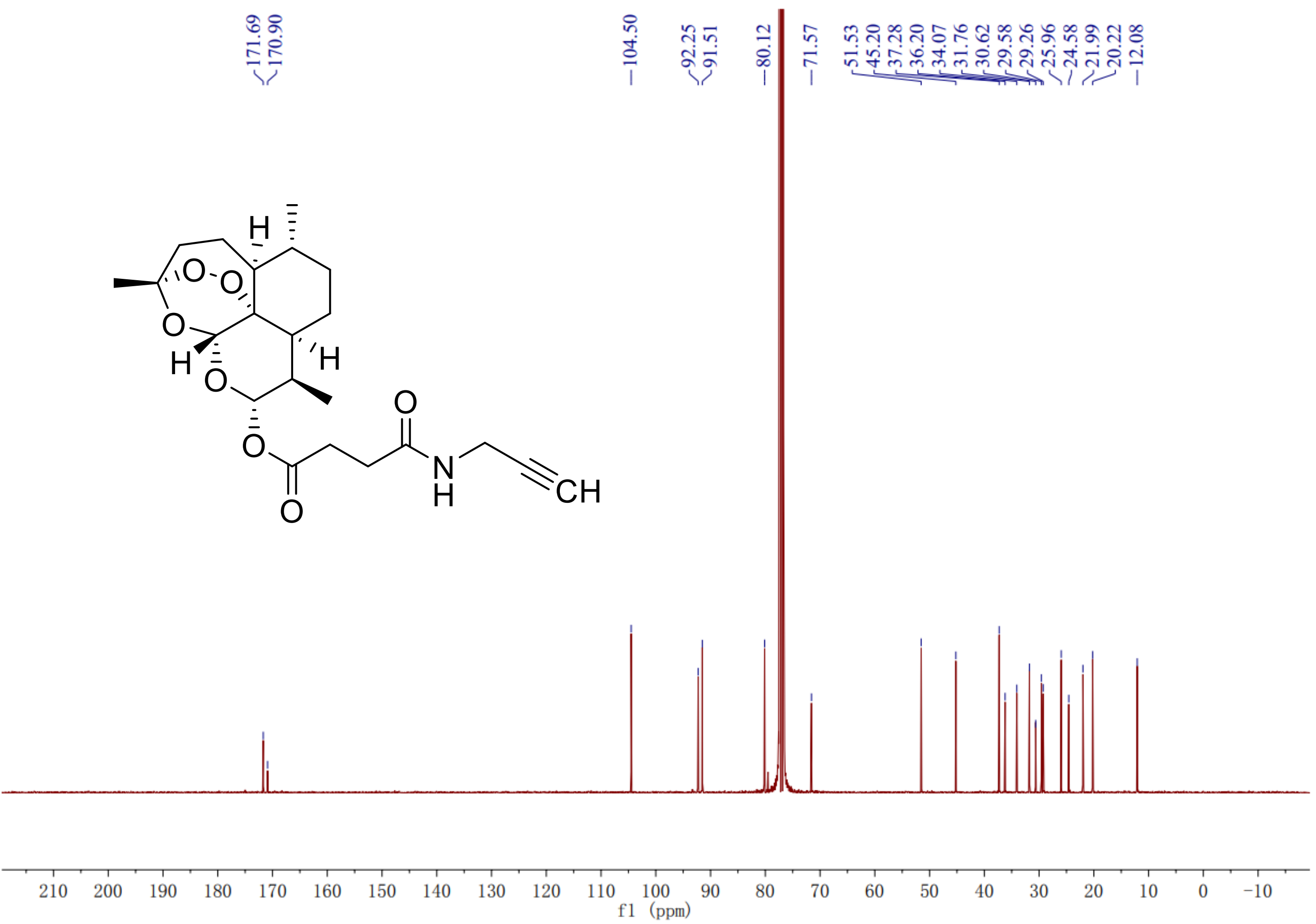

Fig S2 ^{13}C NMR spectrum of PHY-PE in CDCl3

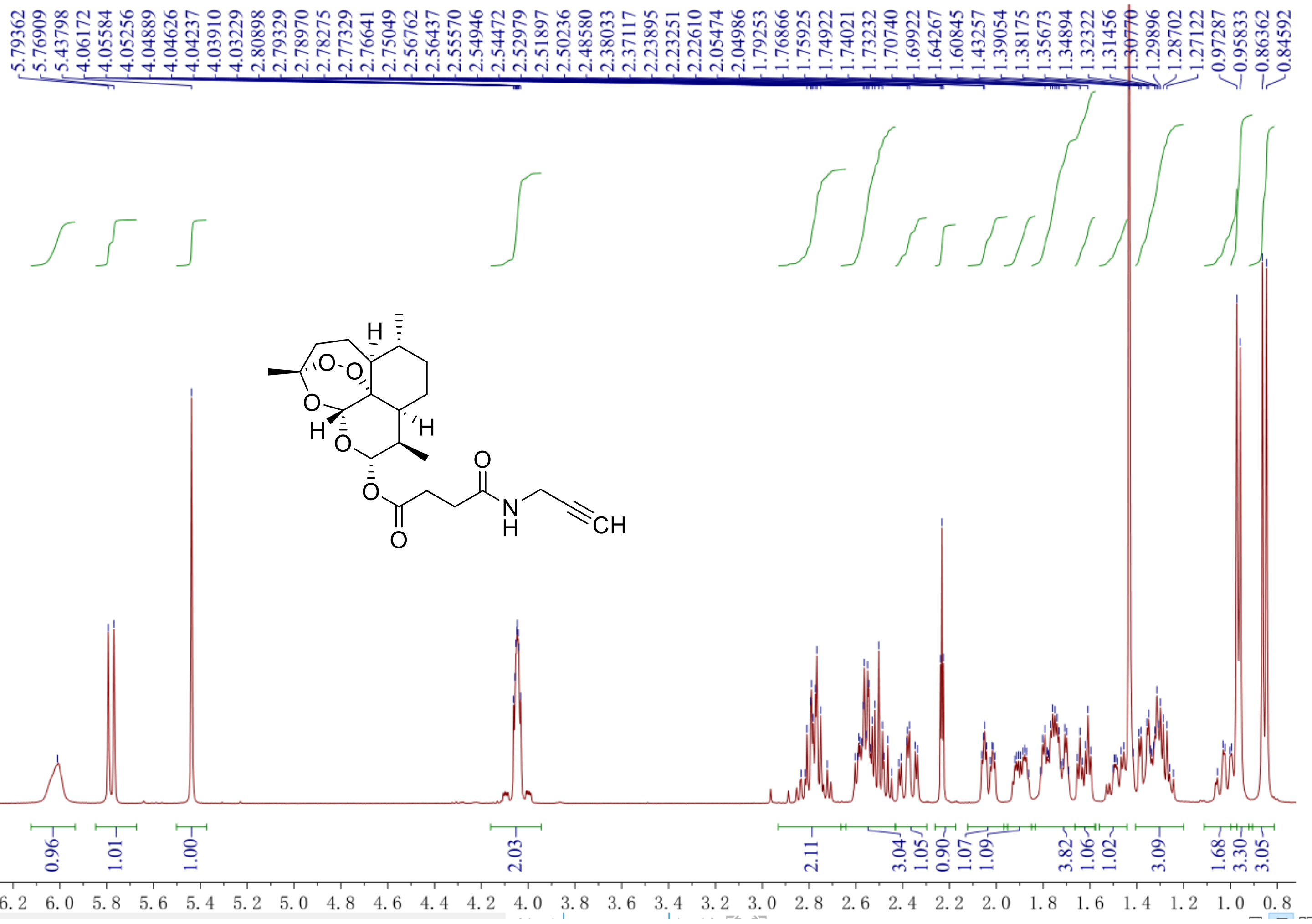

Fig S3 ^{1}H NMR spectrum of PHY-PE in CDCl3

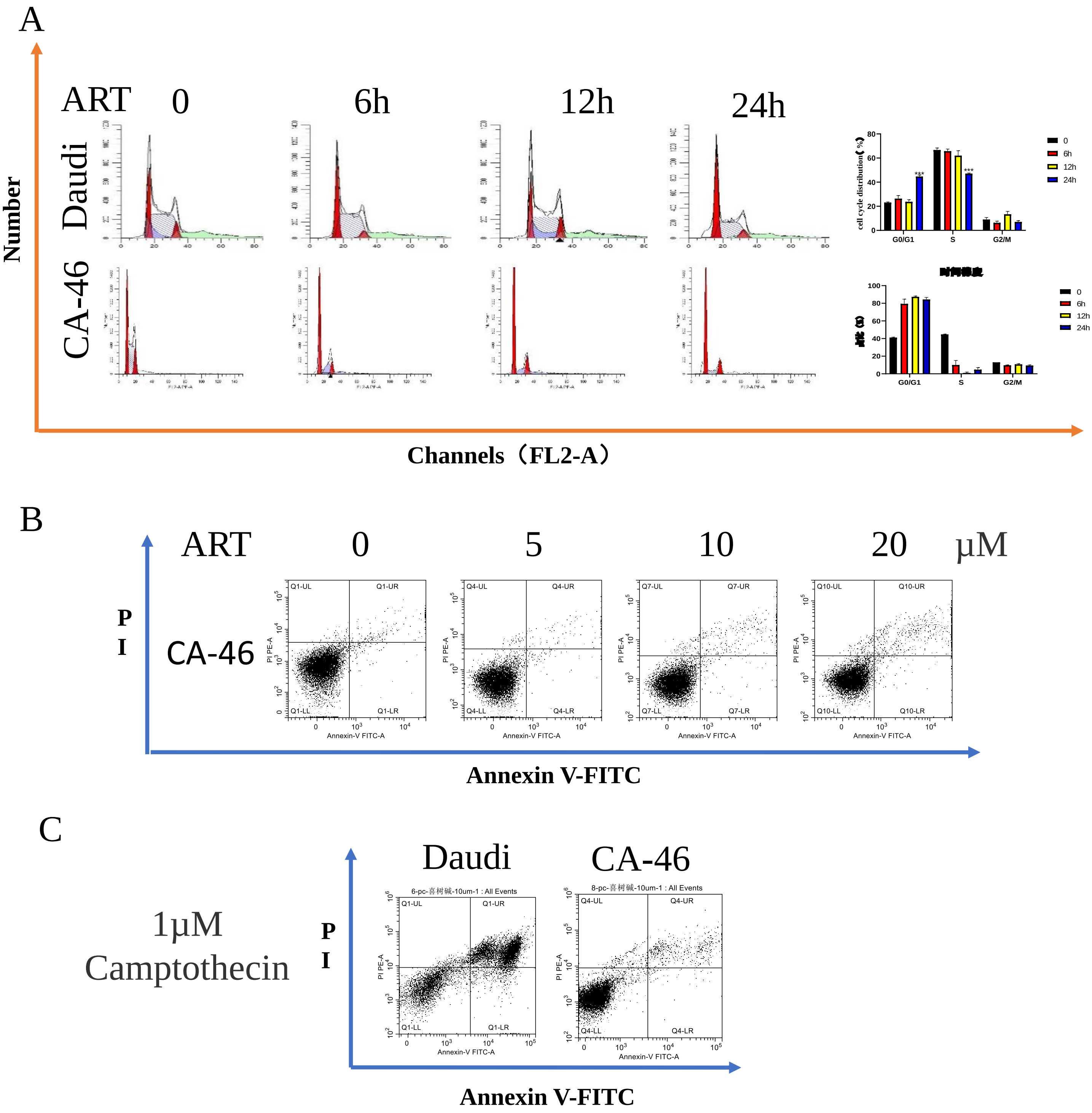

Fig. S4 A. The effect of ART on Daudi and CA-46 cell cycle (Time gradient); B. ART can induce CA-46 apoptosis; C. Apoptosis of Daudi and CA-46 treated by 1μM Camptothecin , The apoptosis rate of CA-46 cells was not significantly increased after ART and Camptothecin treated, indicating that the cells had strong anti apoptotic ability and were not suitable for apoptosis experiments

A

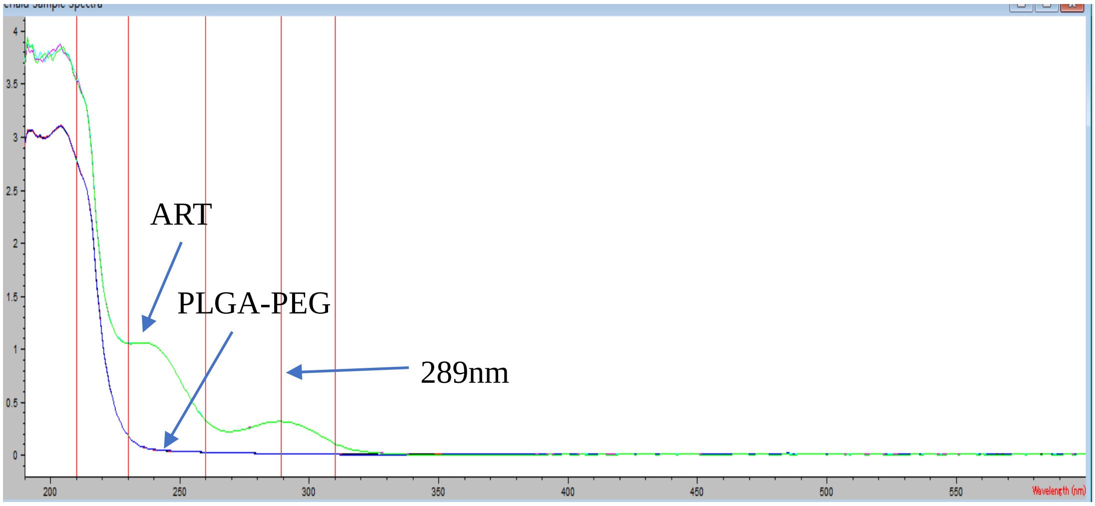

B

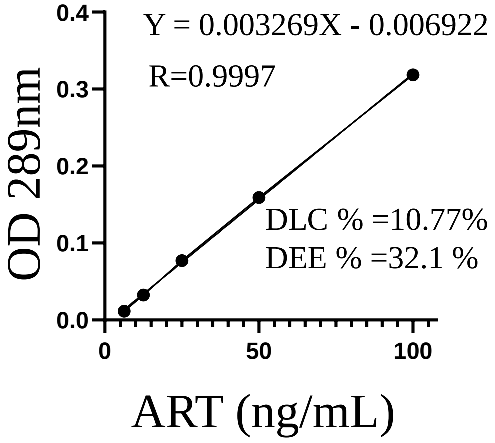

C

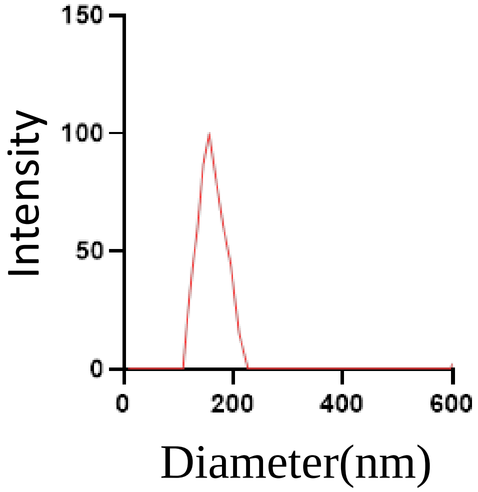

Fig S5 A. ART and PLGA-PEG ultraviolet spectra at 289nm, ART was treated at 50 °C, 1% NaOH, 1h, PLGA-PEG has no UV absorption at 289nm which will not affect the UV absorption of ART. B. Drug loading content (DLC%) and Drug encapsulation efficiency (DEE%) of ART nanoparticles; C. Nanoparticle size.
